# Searching where the treasure is: on the emergence of human companion animal partnership (HCAP)

**DOI:** 10.1007/s10071-020-01467-z

**Published:** 2021-01-12

**Authors:** Ádám Miklósi, Judit Abdai, Andrea Temesi

**Affiliations:** 1 Department of Ethology, Eötvös Loránd University, Budapest, Hungary; 2 MTA-ELTE Comparative Ethology Research Group, Budapest, Hungary

**Keywords:** Human–animal interaction, Animal cognition, Social competence, Caring behaviour

## Abstract

In our view, the discipline, often referred to as human–animal interaction (HAI), lacks a well-defined conceptual framework. It is too narrow both with respect to the animal species investigated and the nature of human–animal interactions studied. So instead, we introduce the term human–companion animal partnership (HCAP) that is not only a better descriptor for most research efforts within HAI but also helps to direct research efforts on an ethological basis. In our approach, ‘companion’ is a function and not a feature of some species. This means that many species had and could have a potential to form mixed social groups with humans if they evolve some capacity of social competence. This view may initiate new comparative research involving a range of species to find out how complex social engagement could be maintained in such hetero-specific social groups based on evolutionary heritage, recent selection and individual experience (socialisation). Our approach emphasises the role of human caring behaviour and social competence in the emergence of a partnership with several species, and thus could also help in setting expectations for welfare and aid in designing artificial companions for specific purposes.

## Introduction

For those who are working in the field of human–animal interaction (HAI), there is nothing peculiar in this label. However, considering it from a biological perspective, HAI as a descriptive name for a scientific discipline is quite unfortunate because taking on face value, much of human life is manifested in interactions with animals. Animals are not just our companions but also we hunt them, we eat them, they are our endo- and ecto-parasites, etc. In other words, the terms ‘animal’ and ‘interaction’ are too general for forming the basis of specific scientific investigations with a particular biological character and interdisciplinary approach.

Over the years, HAI has been referred to in various ways and it is important to realise that the focus of most of the research is actually more specific than implied by the HAI label (Griffin et al. 2019; McCune et al. 2020). Importantly, there have been only a few instances where a definition was provided. For example, Esposito et al. (2011) note that HAI represents “mutual and dynamic relationships between people and animals and the ways in which these interactions may affect physical and psychological health and well-being”. According to this definition, and the majority of (applied) research in HAI should aim to maximise ‘health and well-being’ of humans, similar to some kind of medical treatment (e.g. Mueller et al. 2018).

In this opinion piece, we would like to re-think HAI, based on a biological (ethological) perspective that may also help to connect basic and applied research with practical applications. For us, HAI is not a ‘concept’ (Vitztum 2013) rather a biological phenomenon that has a history (evolution) and also function (fitness) both from the animals’ and humans’ perspectives. The origin of this complex human feature is not yet clear but, for example, according to Wilson (1984), humans show a tendency of being attracted to living organisms (including plants and animals). The so-called ‘biophilia hypothesis’ assumes that humans’ culturally diverse cohabitation with a relatively small range of species has genetic as well as environmental components (Ulrich 1993; but see Kahn 1997).

## Human–companion animal partnership (HCAP)

The ethological approach to non-human and human behaviour offers a very useful framework for scientific investigations that takes into account several related causal factors (Tinbergen 1963). In this way, we can make direct connections to other similar systems, and HAI could be freed from its relatively isolated position being somewhere at the junction of ethology, psychology, pedagogy, education, ethics, etc. without any underlying biological framework.

We suggest that human–companion animal partnership (HCAP) refers to an evolved, mutualistic relationship that unfolded between socialised populations of a non-human species had been selected for social competence (‘companions’) and populations of humans, in which partners routinely spend time within tactile distance and seek regular communicative contact with each other on a daily basis.

The advantage of making a clear distinction between HCAP and HAI is that we have a biologically sound working definition for the former, while all other research efforts with much broader implications involving any kind of animal species could be part of HAI. In short, HCAP becomes a sub-discipline of HAI.

Following the above definition, we identified the following criteria that best describe the interactions, partnership discussed within HCAP.

1. HCAP identifies an ecological relationship, mutualism, as a central concept. This means that the partnership takes place at the level of the community (cf. populations of species) and not at the level of the individual or species. It also follows that benefits and costs should be calculated at the level of the population, and the general assumption is that the partnership evolves if the benefits outweigh the costs.
2. Importantly, ‘companion’ becomes a function, a specific form of social relationship with mutually advantageous character for the participants, rather than a descriptor for certain species (e.g. cats/dogs). Thus HCAP is not particularly interested in transient occurrence of relationships that include animals kept occasionally for a shorter or longer period of time (e.g. snakes) or single individuals where the evolutionary history of the companionship is lacking.
3. The term ‘evolution’ implies that the partnership with humans should have a specific history, involving changes in allele frequencies, perhaps mutations leading to the emergence of specific populations, and perhaps even species (e.g. Albert et al. 2012; McHugo et al. 2019). Thus depending on the evolutionary history of the species involved, there could be a broad variation in the intensity of the partnership (see below).
4. Using ‘interaction’ to ‘partnership’, we aim to express the mutual social interest in both parties in forming inter-species groups (often ‘dyads’). Importantly, such groups should emerge spontaneously at large scale when the populations of the two species have the chance to interact.
5. The reference to ‘social competence’ summarises many aspects of socio-cognitive behaviours that enhance the chances of these animals to live in human proximity, accommodating to some degree to the rules of human social behaviour (Miklósi and Topál 2013; see below). Close-range interactions are typical for humans; thus, similar behaviour is also expected among humans and companion animals.

## The advantageous consequences of establishing HCAP

According to our opinion, we should focus on a phenomenon that has a clear biological (evolutionary) basis. Making a clear argument for HCAP within HAI directs our attention to those instances that are typical. This should also help in eventually coming up with a theory that may explain how these partnerships have emerged, how these partnerships contribute to human well-being in the modern times, what the specific mechanisms are which provide the basis of any (mutual) healing effects, and what kind of novel partnerships may manifest in the future.

Studying HCAP may also reveal a lot about human behaviour that is generally overlooked from other perspectives. The well-being of the non-human partner should also be part of the investigations as well as the possibility of mutually negative effects, including zoonoses or anthroponoses. Finally, HCAP could also inform us about the proposed relationships with non-living autonomous agents, like virtual or real social robots.

HCAP has more specific advantages:

1. There is a potential to develop (arbitrary) quantifiable scales (partnership scales) for various animal species that would reflect the complexity of their partnership with humans. This development could provide a very useful framework for species for which there is a new trend to develop a partnership with humans. Miniature pigs (*Sus scrofa domesticus*) seem to be able to adjust to human social life (Marino and Colvin 2015; Gerencsér et al. 2019), and silver foxes (*Vulpes vulpes*) selected for tameness may also become companions (Trut 1999; Gogoleva et al. 2011). Not surprisingly, the dog (*Canis familiaris*) could be regarded as the core (reference) species for the development of such partnership scales. The long evolutionary connection between dogs and people ensures that dogs possess a broad range of socio-cognitive skills which makes them conforming with little effort to human social rules, that is, they show a high level of social competence (Hare and Tomasello 2005; Topál et al. 2009).
2. How the partner species interact with humans could be the basis to develop comparative studies highlighting the level of adjustment to the human social niche, depending on the evolutionary history and/or the developmental experiences (Miklósi et al. 2005; Gerencsér et al. 2019).
3. HCAP should focus on species that regularly have the chance to get within reaching distance with their human companion and thus can decide the extent of getting involved in mutual social contacts, including tactile interactions. Although we may not want to exclude all animals kept exclusively in a confined location, as their study may not be central to HCAP.
4. Assuming that a natural relationship exists between the partners, the potential negative effects should be also investigated in detail. The probable mutual transmission of diseases, the impairment of physical and mental well-being of both partners should also be investigated, however not only for specific situations (e.g. therapy setting) but also during the whole period of their shared life.
5. Investigating specific cases of interactions between persons and animals, which do not fit the definition, should be peripheral to the main interest of HCAP. Thus, human–crocodile or even human–dolphin interaction may be studied for its own sake but these scenarios represent the few exceptions that have little biological relevance and thus, belong under the general HAI umbrella.

## The human social niche

As far as we know today, humans always took a lead in the establishment of HCAP. Early wolves or dogs may have been attracted by human communities producing much leftovers and edible rubbish but it was humans who eventually decided that dogs were allowed to enter the family circle (for a review see Miklósi 2015). For thousands of years, cats have taken up an important role to keep pests (mice, rats etc.) away from crops, but nowadays they seem to represent the most popular animal partners in human homes (Turner and Bateson 2000). Pigs were also seen as partners in some cultures but their success in becoming partners within a family started with the selective breeding of mini-pigs (Marino and Colvin 2015).

For many populations of these animal species, the human social niche provides the selective environment. To become partners, these animal populations had to adjust to specific features of human social life (Miklósi and Topál 2013). Based on Csányi (2000) and Topál et al. (2009) introduced the concept of the human behaviour complex that refers to a set of human-specific social behaviours that have been altered significantly after the Pan-Homo split. Human behaviour complex defines a three-dimensional space for specific behavioural functions:

1. Sociality involves traits important for living in large, closed social groups, and is manifested in increased social attraction and tolerance, tactile interactions, and mutual sharing resources;
2. Synchronisation increases coordinated interaction among group members facilitated by the emergence of, e.g. empathy and rule-following;
3. Construction, the ability to create new, shared behavioural structures from components that include complex communicative interactions, tool use or culture (Csányi 2000; Topál et al. 2009).

For example, imitative ability involves social interest toward the other, it results in increased behavioural synchronisation among the group mates, and some manifestations can also be seen as a kind of joint construction. Attachment is also a central social feature of human behaviour that contributes a lot toward increased sociality and synchronisation, while it gains little from the constructive skill. As a starting point, it was suggested that the evolution of dogs potentiated this species to adjust to this social niche by displaying many components of the human behaviour complex (Topál et al. 2009). All other non-human companions have faced or will face similar challenges.

## Human caring behaviour and animal partners as family members

Importantly, the existence of the human behaviour complex does not explain on its own why there is a trend on a micro-evolutionary scale in humans to establish partnerships with some non-human species. We can offer a tentative first-hand hypothesis here, mainly to start a discussion about such factors. Humans belong to the few rare mammals in which it is relatively frequent that both sexes share the duties of parenting (Clutton-Brock 1989; Geary 2000; Geary and Flinn 2001). Such caring behaviour should also be selected for in humans because the supporting role of the parents extends over many years. As a consequence, parents do not only supply material resources but continuously provide an intensive social relationship. In addition, human families also are aided by alloparental care from sisters, brothers or grandparents (Geary and Flinn 2001; Del Giudice 2009). In the past, such families may have lived in close social contact forming large groups in comparison to other primates or apes (Dunbar 2012).

Human caring behaviour is controlled by complex mental processes. Importantly, caring behaviour is not only expressed toward infants but also toward adults with whom the donor shares a specific social relationship. Such prosocial behaviour is evoked by a range of sign stimuli (e.g. bodily or facial features, looking behaviour, displays of physical pain or emotional stress, high-pitched vocalisations including crying; e.g. Gračanin et al. 2018) that are characteristic to infants but may also be displayed by adults who are in need or want to direct the attention of the other to themselves (e.g. during courtship). Caring also involves aspects of pedagogy to prepare the infant for future challenges of life. The idea of natural pedagogy (Csibra and Gergely 2011) assumes that the transfer of cultural knowledge is achieved by a range of human-specific behavioural specialisations (both in the parent and the infant) that promote the transmission of complex information that is often beyond the actual mental capacity of the learner.

We assume that selection for increased sociality, extensive caring behaviour and the bias for pedagogy provides the human selective environment in which populations of animals have a chance to evolve and develop close and long-term social (caring) contacts with humans. In addition, there could have been specific periods in human history when the typical extended family structure collapsed for shorter or longer times, and people experienced some kind of social deprivation. Specific cases like this could have offered potential time windows when populations of animals may have had a higher chance to join the population of human families. We should see this as a dynamic process, that is, during specific historical, economic and cultural periods, there could have been an interest or rather a refusal of having companion animals around. Nowadays, many cultures experience social deprivation at the individual level (Hortulanus et al. 2006) that facilitates the craving for animal partners. Thus, companion animals may have a significant role to step in the life of humans when there is shortage or lack of within species social contact (on the positive effects of animal companions on human loneliness see Black 2012; McConnell et al. 2019).

Importantly, in line with the HCAP approach, animals living in such partnerships have the ability to evoke caring behaviour in humans. There are many studies showing an analogy between human caring behaviour toward infants and dogs (e.g. Xu et al. 2015; Gergely et al. 2017) and this is possibly the case with other species as well, like cats or pigs. There is some evidence that interaction between companion dogs and their owners shares similar behavioural and hormonal features as the interaction between mothers and infants (Nagasawa et al. 2009, 2012). In short, caring behaviour has a significant role in maintaining HCAP, and one could hypothesise that selection for participating in such interactions was an important trend in these species. This functional account strongly determines the behavioural mechanisms through which this relationship is maintained, and which can be called upon in cases of need or mental or behavioural malformations (see below).

## The role of tameness for HCAP

While the human behaviour complex and extensive caring behaviour facilitated the emergence of HCAP, in parallel, the non-human species underwent morphological and behavioural changes. It is assumed that tameness represents the first hurdle towards the evolution of partnership with humans. Tameness has often been described as being the manifestation of reduced flight distance (e.g. Agnvall and Jensen 2016). This view is based on the experience of training wild animals and making them tolerant to human presence (e.g. Belyaev 1979).

Being tame presupposes some specific genetic background and experience with the appropriate social environment. Selecting foxes for showing affiliative (approach) behaviour when human strangers offered food to them resulted in genetically tame individuals within 10–15 generations (Belyaev 1979; Trut 1999). Very likely similar processes may have contributed to the tame behaviour in dogs, cats, pigs or other species. But such selection for tameness provides only a potential for a partnership. No individual becomes tame in the absence of socialisation to the presence of humans (Hare and Tomasello 2005). Individual wolves can be tamed (to some extent) (Gácsi et al. 2005; Ujfalussy et al. 2017; Lenkei et al. 2020), but wolves are not tame as a species.

Unfortunately, there are no published studies that show whether selection for tameness (approaching humans) also brings about a specific phenotype (as a by-product) that may show evidence of more complex forms of social interaction or additional selective steps that are needed.

## Socio-cognitive aspects of social competence

The complexity of the partnership depends crucially on the potential of the specific species to match the components of the human behaviour complex. This match could be contingent on the non-human species-specific mental machinery but is also influenced indirectly by the species size, morphology, perceptual skills, or ecology of the ancestors. Relying on a divergent set of visual or acoustic signals in communication may put a species into an advantageous position for interacting with humans. For example, brachycephalic dog breeds display more enduring attention toward humans and are more successful in following human cueing (Gácsi et al. 2009).

We hypothesise that HCAP relies on a more complex social phenomenon (see also Goto et al. 2013), described as social competence (Miklósi and Topál 2013). Taborsky and Oliveira (2012) define social competence as the ability of an individual to optimise the expression of its social behaviour as a function of the available social information. Optimisation means to minimise conflicts that can be achieved by generally following social rules. For example, social competence assumes the ability to form close social relationship with a partner (e.g. attachment), and decreased motivation to occupy a higher social position in the rank order (leading to a more relaxed hierarchical social structure, see Flack and de Waal 2004). It may also include increased social interest (attention) toward the other, the ability to rely on social reference, to use various communicative channels to manage social interaction, and skills of social learning and engagement in cooperative interactions (Miklósi 2015).

The concept of social competence offers a very useful tool by the means of which the socio-cognitive capacities of different non-human species involved in HCAP that can be evaluated in a comparative design (Miklósi and Topál 2013; for more recent reviews pointing in this direction, see, e.g. Marino and Colvin 2015; Marino and Allen 2017; Nawroth 2017). The detailed investigations in a comparative framework may eventually allow the formulation of more specific mechanistic questions of whether high performance or behavioural limitations can be explained by constrains provided by the evolutionary heritage, the state of genetic selection for social competence or depend rather on environmental factors. For example, dog breeds provide a good testing opportunity for investigating variation in social competence because selective breeding has in some cases enforced different aspects of dog–human interaction (Miklósi 2015) but similar scenarios can be envisaged also for cats or horses.

## Partnership as a continuum

Although HCAP focuses on the close relationship between some species and humans, one may deduce other possible scenarios, paradoxically, some of which could be seen as a possible model for the evolutionary situation. For example, some forms of human–animal interaction might have originated in management of livestock. Pigs or rabbits may provide an example for this case. Caring on a daily basis for animals kept in larger group is also a natural situation for HCAP. These individuals may not be considered as family members and social interactions are probably less complex. However, many components of social competence can get activated (Nawroth 2017). The effect of working with such well socialised farm animals, feeding, cleaning or grooming them can provide the experience of close physical contact, nurturing, accomplishing a task or coping with complex situations (see also ‘Green care’ concept Berget and Braastad 2011). Thus, farm-like situations may represent less intensive social scenarios for the partnership spectrum.

Various forms of animal-assisted intervention or therapy represent another extreme form of HCAP with the aim to provide specific physical and mental assistance for children and adult humans in need (e.g. Martin and Farnum 2002; Moretti et al. 2011; O’Haire 2013; Saunders et al. 2017). This kind of interaction also relies in evoking components of the caring behaviour in humans supported by the high level of social competence displayed by the involved individual animals.

This means that along with the variations in social competence, intensity of partnerships with humans can be different among species and during human history and future.

## Animal welfare aspect

In traditional HAI, the focus has been on human well-being, not surprisingly the question of animal welfare emerged only during the last 10 years. Such investigations focused on different types of interventions (e.g. whether animal companions were or could be harmed during various forms of animal-assisted therapies; e.g. McCullough et al. 2018; de Carvalho et al. 2020) or on interactions with livestock (e.g. Jago et al. 1999; Waiblinger et al. 2006).

If the partnership is based on social competence and caring, as predicted by HCAP, then we expect the interaction to emerge as result of mutual interest and in average it should have a positive effect on all partners. In other words, practice must be mutually beneficial to be considered both ethical and effective. For example, less well-socialized companion dogs in an unfamiliar environment would be more inclined to show stress-related avoidance behaviours towards strangers who try to get into close interaction with them (Beerda et al. 2000).

Animal-assisted intervention sessions are highly regulated. Thus, non-human animals (especially dogs) involved in these sessions are selected and trained by exposing them to a number of different situations including meeting strangers (social contact, greeting, handling), playful interaction, and several potential fear- and aggression-evoking stimuli. Thus, these animals have to learn to be socially competent to engage rapidly in an intensive social interaction with a strange or barely familiar person.

## Outlook: digital social technology: friend or foe?

Although the HCAP framework focuses on the partnership between humans and companion animals, it can also be opened to artificial agents as companions which may play an important role in the close future. In recent years, there is an increasing trend for including artificial tools to replace companion animals in certain interventions or therapies (e.g. Probo: Saldien et al. 2008; Paro: Shibata 2004; Huggable: Stiehl et al. 2005). Therapeutic sessions with the Paro robot suggest that the robot has positive effect on the mood, social behaviour and physiological indicators of stress in humans, tested mainly in the elderly (e.g. Inoue et al. 2012; Robinson et al. 2013; see also Marti et al. 2005). Although these results are encouraging, they did not include a thorough investigation on which aspects of the interaction might be important. Sefidgar et al. (2015) suggested that robots should be designed relying on HAI (or rather HCAP) by identifying the key elements that provide benefit for humans in such interactions. Importantly, by changing the embodiment, behaviour and capabilities of the robot, these agents can have different roles in acting as a companion (Miklósi et al. 2017; Miklósi and Gácsi 2012) or in specific therapeutic situations (see Cabibihan et al. 2013). Robots can contribute in the future to improve the health and well-being of humans who cannot participate in animal-assisted therapy or intervention.

## Conclusion

Non-human animals have differential potential to become companion species for humans. During our history, only a handful of species entered the human social niche. However, the possibility is there, especially for those species that are able to evolve some behavioural features that are compatible with human social competence over a few generations. Thus, future research in HCAP should also explore not only the differences of social competence among non-human animals but also the potential of some species to achieve a more intensive partnership with humans and whether other animals may also reach a partnership status.

A parallel process may also lead to artificial companions invading our family life. The behaviour of social robots capable of long-term, meaningful interactions with humans should be modelled based on the behaviour of companion animals (Miklósi et al. 2017). Dogs may serve as a useful model species due to their complex interspecific social competence. Artificial agents may have similar roles as companion animals, if they achieve meaningful levels of social competence.
